# Laryngectomy: Phonation Alternatives and Their Impact on the Quality of Life

**DOI:** 10.7759/cureus.39093

**Published:** 2023-05-16

**Authors:** Ana Rodrigues, Francisco Alves de Sousa, Maria J Casanova, Ana Silva, Telma Feliciano, Susana Vaz Freitas, Ricardo Pinto, João Lino

**Affiliations:** 1 Otolaryngology - Head and Neck Surgery, Instituto de Ciências Biomédicas Abel Salazar, Centro Hospitalar Universitário do Porto, Porto, PRT; 2 Otolaryngology - Head and Neck Surgery, Centro Hospitalar Universitário de Santo António, Porto, PRT; 3 Speech Therapy, Otolaryngology - Head and Neck Surgery, Centro Hospitalar Universitário de Santo António, Porto, PRT; 4 Gastroenterology, Centro Hospitalar Universitário de Santo António, Porto, PRT

**Keywords:** carcinoma of larynx, voice rehabilitation, secel, rehabilitation, phonation, quality of life, esophageal speech, electronic larynx, tracheoesophageal speech, total laryngectomy

## Abstract

Background

The decision to consent to surgery is a life-changing moment. This study addresses the impact of total laryngectomy (TL) on phonation and its effect on the quality of life (QoL) of patients. The primary objective of this cohort study is to compare the alternatives in phonation rehabilitation, and the secondary objective is to identify concurrent predictors of vocal outcomes.

Methodology

To perform a comprehensive analysis, we reviewed data from patients who underwent TL with bilateral radical neck dissection in the Department of Otolaryngology, Head and Neck Surgery at Centro Hospitalar Universitário de Santo António between January 2010 and October 2022. Adult patients who consented to participate in the study and underwent subjective evaluation were included in this study. Data regarding clinical history was primarily collected. Statistical analysis was performed using SPSS version 26 (IBM Corp., Armonk, NY, USA). Different types of vocal rehabilitation formed the subgroups to be compared. An additional analysis was performed for baseline variables collected in the clinical records, and vocal outcomes were measured using the Self-Evaluation of Communication Experiences After Laryngectomy (SECEL) questionnaire. Furthermore, linear models taking SECEL scores as the outcome were developed.

Results

The first search identified a total of 124 patients operated during the study period. In total, 63 patients were alive at the time of the current follow-up, with 61 deaths (49%). Overall, 26 of the 63 alive patients completed the SECEL questionnaire. All patients were male. The mean age at diagnosis was 62.2 ± 10.6 years. The mean age at the time of subjective vocal assessment with the SECEL questionnaire was 66.3 ± 10.4 years. The mean time of follow-up after the initial diagnosis was 4 ± 3.8 years. A statistically significant difference was observed in esophageal speech (ES), which was inferior to other modalities (mean SECEL total score for ES: 46.6 ± 12.2 vs. mean SECEL total score for all other modalities: 33 ± 15.1; p = 0.03). The follow-up time correlated significantly with vocal function, as measured by the SECEL questionnaire (p = 0.013).

Conclusions

The SECEL questionnaire can be a valuable tool to evaluate QoL in laryngectomy patients, given its usefulness in assessing the psychological impact derived from vocal functionality in this group. ES appears inferior to other modalities regarding voice-related QoL.

## Introduction

Although laryngeal cancer is not the most prevalent tumor, it affects a significant portion of patients worldwide. Eastern and South-Central Asia are the most severely impacted regions [1]. According to data, the incidence and mortality of laryngeal cancer are globally higher in men than women [1]. In 2020, Portugal reported 529 new cases and 329 deaths from laryngeal tumors [2]. The larynx controls breathing, protects the airway, and is involved in phonation. Smoking is the primary risk factor. Other known risk factors are alcohol abuse, radiation exposure, and premalignant lesions (from reflux or human papillomavirus) [3]. Chemotherapy, radiation, and surgery are the most frequently used treatments. The treatment decision is influenced by the tumor’s characteristics and the patient’s behavior, performance status, and socioeconomic circumstances [4].

Total laryngectomy (TL) usually involves the removal of all of the thyroid and cricoid cartilage, the arytenoid cartilage, the epiglottis, the hyoid bone, and the prelaryngeal muscles [3]. The pharyngeal tube is closed using a horizontal or T-shaped suture [3]. The cut end of the trachea is sutured to the skin of the neck and an end stoma is created [3]. The removal of the larynx has profound consequences for a patient [3]. The separation of the airway from the mouth, nose, and esophagus leads not only to the loss of the ability to speak but also to the separation of the nasal and pharyngeal segments from the lower airways, as well as the loss of the air conditioning mechanism and active smelling [3]. Patients must learn to cope with a tracheostoma and the associated disadvantages [3].

Billroth performed the first TL in Vienna, Austria, in 1873. The effects of laryngectomy-related morbidity on voice production were already a cause of worry at the time [3]. Hence, Gussenbauer developed the first mechanical larynx, which comprised a tracheostomy and pharyngeal cannula [3]. The prognosis and patient survival improved dramatically as a result of these techniques which revolutionized the treatment of tumors of the larynx [3]. Researchers continued to develop several phonation options in the 20th and 21st centuries, and alternatives such as tracheoesophageal prosthesis (TEP), electronic larynx (EL), and esophageal speech (ES) were proposed [3]. Nevertheless, surgery continues to be very intrusive, with a significant impact on everyday life and making rehabilitation a lifetime endeavor [5].

TL, occasionally combined with adjuvant radiotherapy and/or chemotherapy, is the recommended course of treatment in many cases of advanced laryngeal cancers [3]. The decision to consent to surgery is a significant turning point in patients’ lives. Post-laryngectomy quality of life (QoL) has long been associated with the ability to regain communication skills [6]. Nowadays, the most common forms of rehabilitation are ES, TEP, and EL [1,3,4].

The first aim of this study is to assess and compare the impact of different speech rehabilitation alternatives on laryngectomy patients’ QoL. The second aim is to identify and predict factors of vocal outcomes within the study cohort.

## Materials and methods

Sample enrollment and evaluation

To perform a comprehensive analysis, we reviewed data from patients who underwent TL with bilateral radical neck dissection at the Department of Otolaryngology, Head and Neck Surgery of Centro Hospitalar Universitário de Santo António between January 2010 and October 2022. Of those, only living patients were selected. Data including sex, alcohol and tobacco abuse, date of diagnosis, concurrent comorbidities, tumor location (supraglottic, glottic, or subglottic), TNM staging (classification of malignant tumors), adjuvant therapy (radiation or chemotherapy), time of follow-up, and type of vocal rehabilitation were primarily collected. Finally, only adult patients who consented to participate in the study and who underwent subjective evaluation were included in this study.

Subjective measurements (Self-Evaluation of Communication Experiences After Laryngectomy questionnaire)

From October 2022 to March 2023, the previously selected patients were recruited, and vocal outcomes were measured by the Self-Evaluation of Communication Experiences After Laryngectomy (SECEL) questionnaire. The SECEL questionnaire was specifically developed for assessing communication dysfunction in patients with laryngectomies and has demonstrated adequate psychometric properties [7]. It was also validated for European Portuguese [8]. The questionnaire comprises 35 items that explore communication experiences and dysfunction (see Appendices). In total, 34 elements are grouped into three subscales. The initial subscale, General (five items), indicates overall attitudes toward relaxation or calmness, as well as recognition of the illness and therapy. The second subscale, Environmental (14 questions), focuses on how the patient perceives his/her voice in various settings. The third subscale, Attitudinal (15 questions), measures attitudes toward speech, as well as thoughts regarding self-assessment and perception of others. Each item is scored on a four-point scale ranging from 0 (never) to 3 (always), with the latest 30 days addressed. Subscales and the total scale are scored using basic addition. As a result, the summary scale scores range from 0 to 15 for General, 0 to 42 for Environmental, 0 to 45 for Attitudinal, and 0 to 102 for Overall. A higher score indicates a worse perception of functional communication. Finally, the 35th item is a categorical item, including three response options, namely, Yes/More/Less, and is not scored.

Ethics

Informed consent was obtained from all patients. The study was approved by the local ethics committee (approval number: 181-DEFI/184-CE), and the study design complied with the ethical standards of the Declaration of Helsinki.

Statistical analysis

Statistical analysis was performed using SPSS version 26 (IBM Corp., Armonk, NY, USA). In the descriptive analysis, categorical variables are presented as percentages, and continuous variables are presented as means and standard deviations, or medians and interquartile ranges for variables with skewed distributions. Normal distribution was checked using skewness and kurtosis. A bivariate analysis regarding baseline variables collected in the clinical records versus vocal outcomes measured by the SECEL questionnaire was undertaken. The associations were analyzed using either the independent t-test (parametric analysis) or the Mann-Whitney test (non-parametric analysis) depending on the tests for normality, Pearson chi-square/Fisher’s tests (95% confidence intervals) for categories, and Spearman’s test for continuous variables. Finally, general linear models taking SECEL scores as the outcome were developed. All reported p-values are two-tailed, with a p-value ≤0.05 indicating statistical significance.

## Results

Study population

The first search identified a total of 124 patients who had undergone an operation during the study period. Of those, 63 patients were still alive at the time of the current follow-up, with 61 deaths (49%). In total, 26 of the 63 living patients completed the SECEL questionnaire (41.3%) and were included in the final sample.

All patients (100%) were male. The mean age at diagnosis was 62.2 ± 10.6 years (range = 38-83 years). The mean age at the subjective vocal assessment with the SECEL questionnaire was 66.3 ± 10.4 years (range = 46-87). The mean time of follow-up after the initial diagnosis was 4 ± 3.8 years. Other relevant descriptions of the population characteristics are displayed in Table 1.

**Table 1:** General descriptive analysis of registered relevant variables. SD = standard deviation; IQR = interquartile range (25-75) a: Refers to patients who did not successfully achieve any source of vocal rehabilitation despite attempts (including the inaptitude to use ELS). b: Primary (TES1) refers to tracheoesophageal prosthesis placement in the same operating time as the laryngectomy procedure. Secondary (TES2) refers to tracheoesophageal voice prosthesis placement at a different (later) operation with that specific purpose.

Categorical variables	Frequency (%)	Continuous variables	Mean/Median (SD, IQR*)
Primary tumor location		Age at diagnosis (years)	62.2 (10.6)
Supraglottic	65.4	Age at SECEL (years)	66.3 (10.4)
Glottic	34.6	Follow-up time (years)	4 (3.8)
Subglottic	0	SECEL questionnaire (question)	
TNM staging		One	2 (1-3)
Stage II	11.5	Two	2.5 (2-3)
Stage III	34.6	Three	1.5 (0-3)
Stage IV	53.8	Four	3 (3-3)
Risk factors		Five	1 (0-3)
Alcohol abuse	61.5	Six	0 (0-2)
Smoking	88.5	Seven	1 (0-1.25)
Adjuvant therapy		Eight	3 (1-3)
None	26.9	Nine	1 (1-2)
Radiotherapy	34.6	Ten	3 (1-3)
Chemotherapy	3.8	Eleven	2 (1-3)
Radiotherapy + chemotherapy	34.6	Twelve	1 (0-3)
Type of vocal rehabilitation		Thirteen	0 (0-1)
None ^a^	11.5	Fourteen	3 (1-3)
		Fifteen	3 (1-3)
Esophageal speech (ES)	38.5	Sixteen	2.5 (0.75-3)
		Seventeen	1 (0-3)
Electrolaryngeal speech (ELS)	19.2	Eighteen	1 (0-3)
		Nineteen	3 (0.75-3)
Tracheoesophageal speech (TES)	30.8	Twenty	0 (0-1)
Primary (TES1)^ b^	7.7	Twenty-one	0 (0-1)
Secondary (TES2) ^b^	23.1	Twenty-two	1 (0-2)
Question 35 SECEL		Twenty-three	0.5 (0-2)
Yes	11.5	Twenty-four	0 (0-1.25)
More	3.8	Twenty-five	1 (0-3)
Less	84.6	Twenty-six	0 (0-0.25)
Other comorbidities		Twenty-seven	0 (0-1)
Hypertension	38.5	Twenty-eight	0 (0-1)
Diabetes	30.8	Twenty-nine	0 (0-1)
Dyslipidemia	38.5	Thirty	1 (0-1)
Sleep disturbance	38.5	Thirty-one	0 (0-0)
Gastrointestinal	19.2	Thirty-two	0 (0-0)
Previous neoplasia	11.5	Thirty-three	0 (0-1)
Neurologic	7.7	Thirty-four	0 (0-1)
Cardiac	11.5	SECEL scores	
Pulmonary	23.1	General	9.6 (3)
Autoimmune	7.7	Environmental	20.4 (11.1)
Immunosuppression	15.3	Attitudinal	10.1 (7.6)
Depression	11.5	Total	40.1 (16)

Types of vocal rehabilitation: impact on vocal outcomes

Three patients were excluded from further analysis for not using any phonation alternative. When comparing different modalities of successful vocal rehabilitation, we observed a statistically significant difference regarding ES, which was inferior to other modalities (mean SECEL total score for ES: 46.6 ± 12.2 vs. mean SECEL total score for all other modalities: 33 ± 15.1, p = 0.03). When analyzing subscores, this was particularly noted in the environmental domain (mean environmental SECEL subscore for ES: 24.4 ± 7.7 vs. mean environmental SECEL subscore for all other modalities: 14.6 ± 10.1, p = 0.019). No significant differences were observed regarding the general SECEL subscore between ES and other groups (mean general SECEL subscore for ES: 10.4 ± 3.1 vs. mean general SECEL subscore for all other modalities: 9.9 ± 2.6, p = 0.690). Likewise, no significant differences were observed regarding the attitudinal SECEL subscore between ES and all other modalities (mean attitudinal SECEL subscore for ES: 11.8 ± 8.6 vs. mean attitudinal SECEL subscore for all other modalities: 8.5 ± 6, p = 0.285).

When the same statistical technique was employed for TES against all other modalities, no significant differences were observed (mean SECEL total score for TES: 39.1 ± 20.5 vs. mean SECEL total score for all other modalities: 38.8 ± 11.6, p = 0.962; mean general SECEL subscore for TES: 9.7 ± 3.6 vs. mean general SECEL subscore for all other modalities: 10.4 ± 2.1, p = 0.578; mean environmental SECEL subscore for TES: 18.6 ± 12.9 vs. mean environmental SECEL subscore for all other modalities: 19.1 ± 8.6, p = 0.917; and mean attitudinal SECEL subscore for TES: 10.9 ± 9.8 vs. mean attitudinal SECEL subscore for all other modalities: 9.3 ± 5.4, p = 0.617).

When the same statistical method is employed for electronic larynx speech (ELS) against all other modalities, no significant differences were observed (mean SECEL total score for ELS: 35.6 ± 24.2 vs. mean SECEL total score for all other modalities: 39.8 ± 12.6, p = 0.595; mean general SECEL subscore for ELS: 9.4 ± 2.7 vs. mean general SECEL subscore for all other modalities: 10.3 ± 2.8, p = 0.521; mean environmental SECEL subscore for ELS: 17.2 ± 15.5 vs. mean environmental SECEL subscore for all other modalities: 19.3 ± 8.8, p = 0.689; and mean attitudinal SECEL subscore for ELS: 9 ± 9.5 vs. mean attitudinal SECEL subscore for all other modalities: 10.1 ± 6.8, p = 0.808). Other relevant one-to-one comparisons between vocal rehabilitation modalities are displayed in Table 2.

**Table 2:** Matched comparison between vocal rehabilitation modalities. SD = standard deviation; ELS = electronic larynx speech; TES = tracheoesophageal speech; ES = esophageal speech; NS = no speech

Variable	ES (n = 31)				ELS (n = 22)			
	Mean (±SD)	P-value against			Mean (±SD)	P-value against		
		ELS	TES	NS		ES	TES	NS
Age at diagnosis (years)	63.9 ± 11	0.310	0.886	0.769	55.4 ± 15.3	0.310	0.334	0.242
Age at SECEL (years)	67.1 ± 10.1	0.381	0.669	0.903	60.2 ± 14.7	0.381	0.271	0.430
Follow-up time (years)	10 ± 3.2	0.442	0.175	0.122	4.8 ± 3.6	0.442	0.630	0.130
SECEL total	46.6 ± 12.2	0.254	0.005	0.002	35.6 ± 24.2	0.254	0.644	<0.001
SECEL general	10.4 ± 3.1	0.534	0.913	0.047	9.4 ± 2.7	0.534	0.593	0133
SECEL environmental	24.4 ± 7.7	0.243	0.002		17.2 ± 15.5	0.243	0.488	
SECEL attitudinal	11.8 ± 8.5	0.596	0.235		9 ± 9.5	0.596	0.812	
	TES (n = 13)				NS (n = 3)			
	Mean (±SD)	P-value against			Mean (±SD)	P-value against		
		ES	ELS	NS		ES	ELS	TES
Age at diagnosis (years)	63.2 ± 7.9	0.886	0.334	0.642	65.3 ± 5.5	0.769	0.242	0.642
Age at SECEL (years)	69.1 ± 9.5	0.669	0.271	0.549	66.3 ± 5	0.903	0.430	0.549
Follow-up time (years)	5.9 ± 4.1	0.175	0.630	0.013	1 ± 0.5	0.122	0.130	0.013
SECEL total	31.4 ± 6.9	0.005	0.644	<0.001	62.1 ± 22.5	0.002	<0.001	<0.001
SECEL general	10.3 ± 2.7	0.913	0.593	0.042	6 ± 2.6	0.047	0.133	0.042
SECEL environmental	13 ± 5.3	0.002	0.488		32 ± 8.2	0.002	<0.001	<0.001
SECEL attitudinal	8.1 ± 3.2	0.235	0.812		19 ± 10.3	0.047	0.015	0.02

Finally, no significant associations were found between the answer to question 35 and any factor (p > 0.05 for every studied variable). Likewise, no significant differences were found regarding tumor location (glottic vs. supraglottic) and SECEL total score (p = 0.235). No significant differences were found between primary (TES1) and secondary (TES2) rehabilitation concerning SECEL total score (p = 0.652).

Other potential predictors of vocal outcomes

A significant inverse correlation was found between follow-up time and SECEL total score (p = 0.013); hence, increased follow-up time was associated with better perceived vocal function. A similarly significant inverse correlation existed between environmental subscores (p = 0.005). The two other subscores did not reveal any significant correlation with follow-up time (general: p = 0.638; attitudinal: p = 0.199).

No association was found between the age of diagnosis and SECEL total score (p = 0.743). Likewise, no associations were found between the age of diagnosis and any of the SECEL subscores (general: p = 0.884; environmental: p = 0.716; attitudinal: p = 0.907). Age at SECEL did not correlate with the SECEL total score (p = 0.531). Similarly, no associations were found between age at SECEL and any of the SECEL subscores (general: p = 0.825; environmental: p = 0.576; attitudinal: p = 0.525).

**Figure 1:**
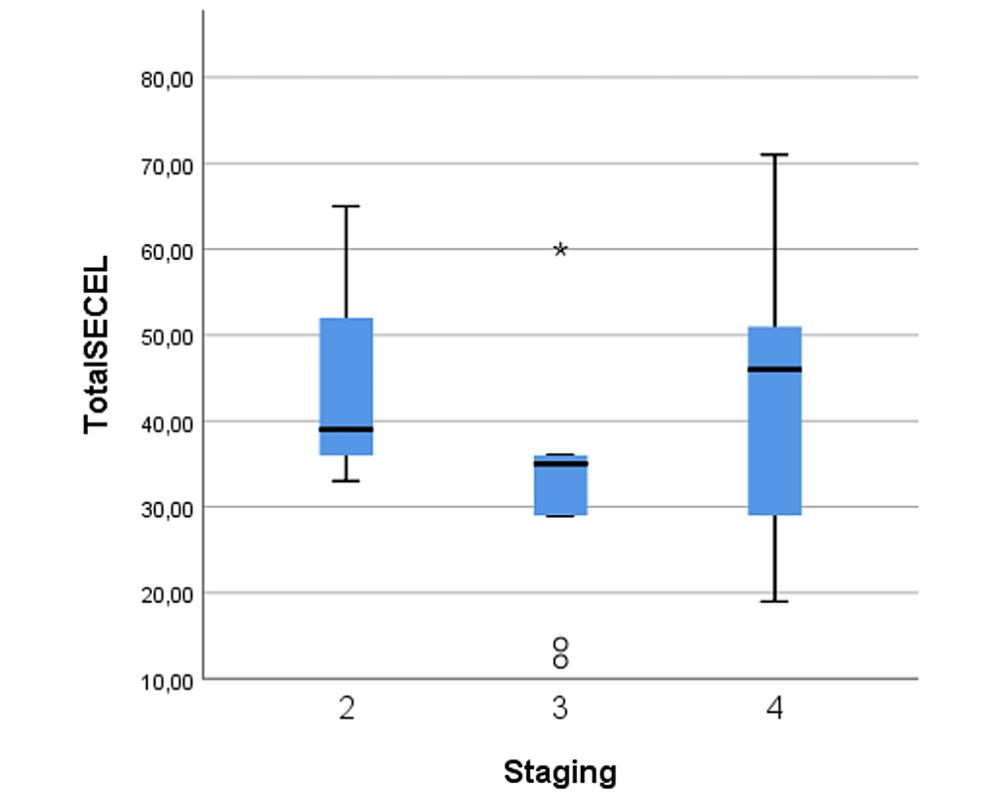
Box plot of mean SECEL total scores among different staging groups. SECEL = Self-Evaluation of Communication Experiences After Laryngectomy

No significant correlations were found between TNM staging and vocal outcomes measured by SECEL total score (p = 0.151), as displayed in Figure 1. Likewise, no significant differences were observed between different adjuvant therapy groups regarding vocal outcomes (neither SECEL total nor subscores, p > 0.05 in all matched comparisons from independent t-test). Furthermore, tumor location was not associated with significant differences in the SECEL total score (glottic mean SECEL total score: 37.1 ± 14.1 vs. supraglottic mean SECEL total score: 45.9 ± 18.6, p = 0.235). Regarding tobacco or alcohol abuse, there was no association between these factors and SECEL outcomes (p > 0.05). The same was observed concerning comorbidities, without any particular comorbidity relating to SECEL outcomes (p > 0.05 for all measured comorbidities).

Multivariate analysis for vocal outcome predictors

**Figure 2:**
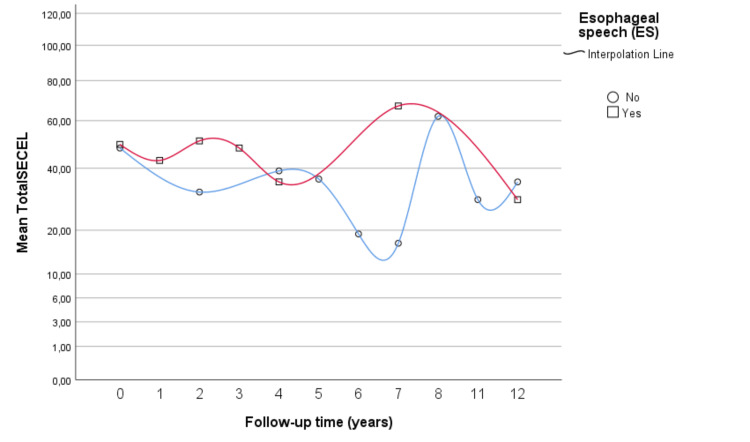
Mean SECEL total scores matched to follow-up time: ES versus other modalities. SECEL = Self-Evaluation of Communication Experiences After Laryngectomy; ES = esophageal speech

This section departs from the above-identified variables correlating significantly with SECEL scores, namely, type of vocal rehabilitation and follow-up time. A linear regression model was calculated to predict SECEL total score based on the vocal rehabilitation subgroup as independent variables (ES or other modality). A significant regression equation was found (F (1,21) = 5.394, p = 0.03), with an R^2^ of 2.204. The fitted model equation was the SECEL total score = 33 + 13.6x (x = 1 if ES or x = 0 if another modality). Using the same method but taking follow-up time as an independent variable resulted in a non-significant model (p = 0.099). Moreover, when both independent variables were accounted for simultaneously in the same regression, the result was a non-significant model (vocal rehabilitation modality: p = 0.74 and follow-up time: p = 0.250). Figure 2 displays the findings related to the time and type of speech rehabilitation.

## Discussion

Being unable to produce a voice is a major life-altering event that significantly lowers QoL. Our findings suggest that ES is less effective than other rehabilitation methods concerning QoL-related vocal outcomes. This is consistent with the majority of recently reported literature on post-laryngectomy phonation options [9-12]. However, some studies, including the one by Mourkarbel et al., assert that there is no statistically significant difference in outcomes between ES and TEP [13]. In contrast, Salturk et al. reported that patients with ES showed better outcomes than those with EL or TES [14]. Chone et al. [15] concluded that primary tracheoesophageal punctures had a higher success rate than secondary ones. However, no discernible differences between primary (TES1) and secondary (TES2) TES rehabilitation were found in our study. Although TES is considered the gold standard and produces better vocal results, it has drawbacks that are not present with ES rehabilitation, including leakage, biofilm formation, infections, and a greater risk of pneumonia [3]. Therefore, many factors, including the patient’s opinion, must be taken into account when choosing between the available options [3].

The secondary goal of this study was to investigate the concurrent baseline predictors of vocal outcomes in laryngectomy patients. We found that follow-up time may have a positive impact on vocal function. This may relate to the learning curve on how to produce adequate voice and communication. Longer rehabilitation time and longer voice usage in daily life may help explain these findings. Additionally, QoL for these patients appears to be significantly impacted by rehabilitation [9].

Regarding other potential baseline predictors, no significant correlations were found between SECEL outcomes and various adjuvant therapy groups, tumor locations, risk factors (tobacco or alcohol abuse), or comorbidities. Furthermore, no significant correlations were identified between TNM staging and vocal outcomes.

From the patients’ viewpoint, TL may imply mutilation, given that there is an impact on their voice, breathing, swallowing, and even taste and smell [3]. It is a life-changing moment with a considerable impact on QoL. QoL is influenced by one’s physical condition, degree of independence, social connections, external circumstances, and personal convictions. A study on the QoL of laryngectomy patients (both partial and total) revealed that the social and emotional functions of the group of patients who underwent TL were most adversely impacted [16]. Additionally, results suggested that all parameters for this group worsened overall [16]. Voice quality is undoubtedly one of the factors that affect the quality of life, but other factors include changes in the body’s appearance, communication, mental state, and social interactions after TL [11]. Body appearance in women undergoing TL appears to pose a higher impact than in men [17]. This lowering of QoL is largely modulated by stigmatization, as a large portion of these patients are unable to keep their jobs and occasionally exhibit aggressive behavior toward those who do not comprehend them [17]. Studies have shown that patients with head and neck cancer may exhibit high rates of psychiatric affection. This may be linked to the disease itself or treatment, which makes the assessment of how phonation has an impact on QoL even more crucial [3].

This study has multiple limitations. The study had a small sample size. Only 63 of the 124 patients who underwent surgery during the study period were alive at the time of the current follow-up, which meant that nearly half (49%) of the study sample had passed away. In addition, only about 41% of patients who were alive completed the SECEL questionnaire. In our experience, it is difficult to deal with TL patients who frequently do not want to reveal their frailties. Besides, communicational limitations may also pose a significant challenge in answering the questionnaire. In addition, the assessment of how it affects QoL depends on various factors that were not directly assessed, such as financial stability, degree of independence, social relationships, environmental factors, and personal views [18]. Therefore, further studies with larger sample sizes and wider assessments of QoL domains are required.

This study has some strengths. It is the first research to compare different phonation methods using the SECEL questionnaire. This tool can help healthcare professionals create a rehabilitation plan more targeted to the patient’s needs. SECEL is helpful in identifying the psychological impact and QoL in TL patients. Caring, informing, and accompanying are key to any treatment.

## Conclusions

Patients who have undergone TL must relearn how to interact with others. TL poses many challenges ranging from alterations in voice production, breathing, and swallowing to emotional and social impingement. The post-TL adaptation is always modulated by the effects of physical appearance on self-esteem and personal views.

Rehabilitation has a significant impact on the QoL of TL patients. SECEL questionnaire can be a helpful tool to assess TL patients’ QoL because it measures the psychological effects of vocal functionality on this population. In this study, we found that TES leads to the best outcomes concerning SECEL scores for voice-related QoL and that ES appears to be inferior to other rehabilitation modalities. Importantly, follow-up time correlated positively with QoL vocal outcomes measured by the SECEL questionnaire.

## Figures and Tables

**Figure 3:**
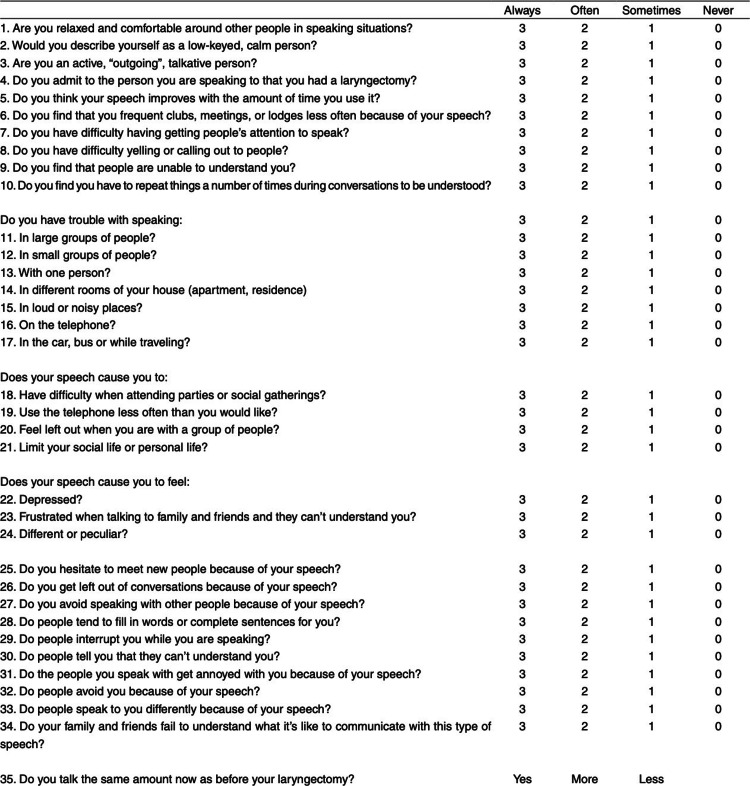
Original Self-Evaluation of Communication Experiences After Laryngectomy questionnaire (English-language version). The questionnaire was developed by Blood GW [7].

**Figure 4:**
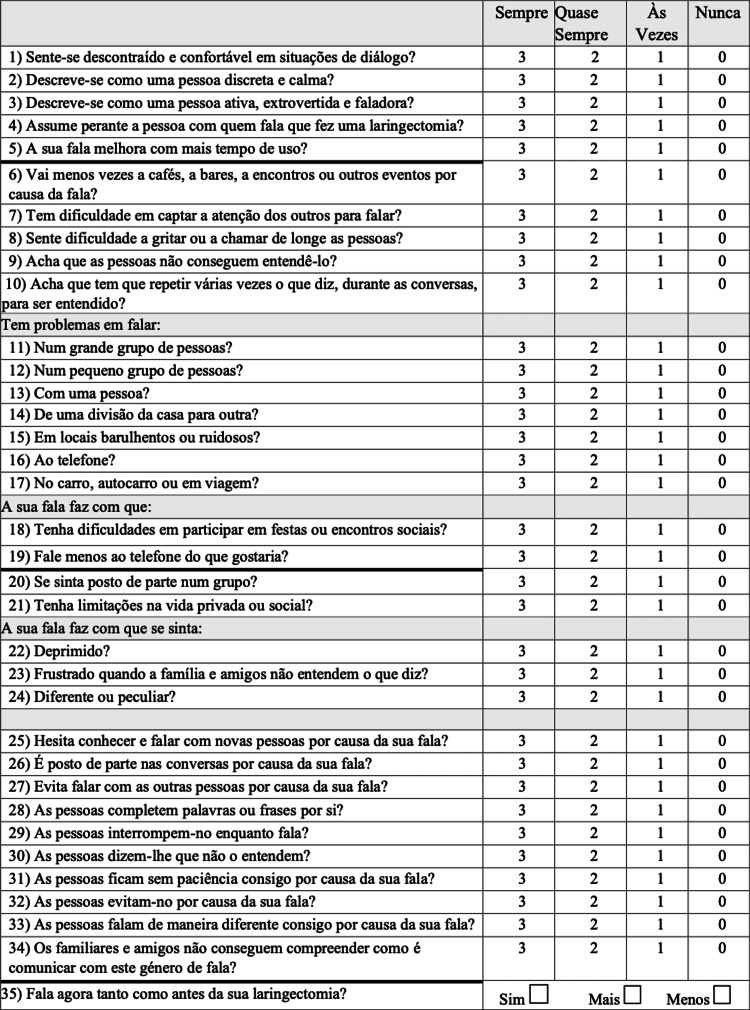
The translated version of the Self-Evaluation of Communication Experiences After Laryngectomy questionnaire in European Portuguese. Originally translated by Antunes S [8].
